# Supplementary material for: Evaluation of residue management practices on barley residue decomposition
Source: PLoS One. 2020 May 13;15(5):e0232896. doi: 10.1371/journal.pone.0232896 (PMC7219745; doi:10.1371/journal.pone.0232896)

**S1 Fig.** **Calibration and prediction of the first order decay constant for residue decomposed under barley residue decomposition study conducted in laboratory at 25-30°C.**


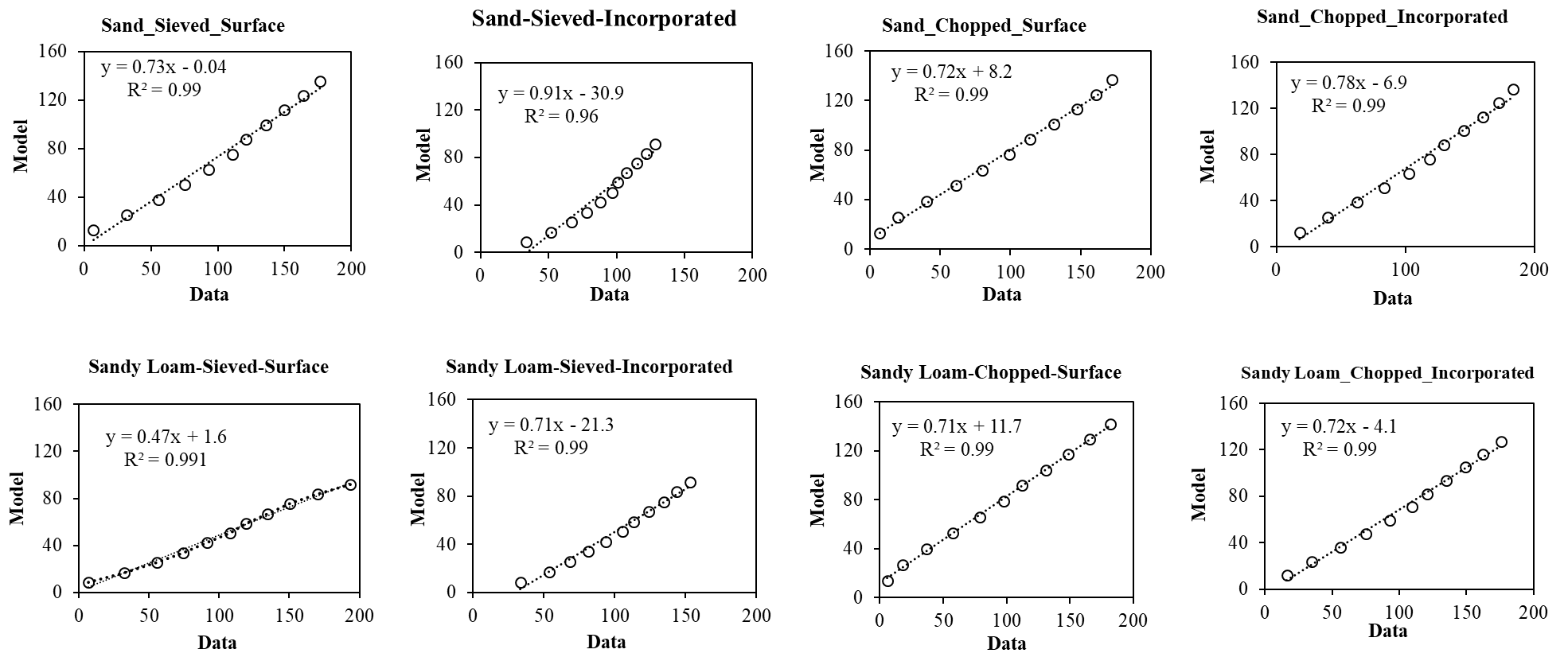

Supplement: S1 Fig — (DOCX) [file pone.0232896.s002.docx]
